# Supplementary material for: Analysis of cathepsin S expression in gastric adenocarcinoma and in Helicobacter pylori infection
Source: PLoS One. 2022 May 26;17(5):e0268836. doi: 10.1371/journal.pone.0268836 (PMC9135267; doi:10.1371/journal.pone.0268836)
Supplement: Attachment — Submitted filename: Response_to_reviews.docx [file pone.0268836.s001.docx]

**Detailed Response to Reviewers**

Manuscript Ms# PONE-D-21-36174 entitled “Analysis of cathepsin S expression in gastric adenocarcinoma and in Helicobacter pylori infection”.

#### Dear Editors and Reviewers,

We would like to thank the reviewers for the constructive criticism regarding our manuscript entitled “Analysis of cathepsin S expression in gastric adenocarcinoma and in *Helicobacter pylori* infection”.

A point-by-point response to the reviewers’ comments was included below. The alterations performed in the manuscript are highlighted in yellow. All authors have read and approved the revised version of this manuscript. The authors have no financial interest and no conflicts of interest to disclose.

We look forward to hearing from you concerning on the suitability of the revised manuscript for publication.

Yours sincerely,

The corresponding author

**# Reviewer 1**

In this study, Costa et al. have evaluated the expression of cathepsin S in gastric cancer and H. pylori infection. The study has reported excellent findings; however, before publication, several points deserve the authors’ attention.

**Answer: We thank the reviewer for the comments and constructive criticism. We tried to comply with all the suggestions made**

Major comments

Abstract

1. Results. In the gastritis group, CTTS expression was significantly higher in patients with positive test for H. pylori than negative test for H. pylori: 87.5% and 38.5%, respectively (p<0.001).

**Answer: We have adjusted the referred sentence.**

Introduction

1. Paragraph 1. Move…… (cathepsin B, H, K, L, and S) after…….at least five (………) have ……..

**Answer: We have adjusted the sentence.**

2. Paragraph 2. Epidermal growth factor (EGF), vascular endothelial growth factor (VEGF), and tumor growth factor-beta (TGFβ),………….

**Answer: We have adjusted the sentence according to what was indicated.**

3. Paragraph 2. Activated cathepsins hydrolyze growth factors, EGF, VEGF, and TGFβ, promoting cancer cell proliferation and angiogenesis,……….. The sentence is a bit confusing, after hydrolysis if they are destroyed then how cell proliferation occurs. The hydrolysis renders maturation of the EGF, VEGF, and TGFβ, then promotes the cancer cell proliferation.

**Answer: Cathepsins hydrolyze these growth factors, activating them and promoting cell proliferation. (Chen S, Dong H, Yang S, Guo H. Cathepsins in digestive cancers. Oncotarget. 2017;8(25):41690-41700. doi:10.18632/oncotarget.16677).**

**We have rephrased the sentence to turn it clearer.**

Materials and Methods

1. Study design. Paragraph 1, line 7. ….. and other with a negative result for H. pylori (n=25).

**Answer: We have adjusted the sentence.**

2. For the sake of understanding it is better to name the tables in immunohistochemistry as Table 1, 2, and 3 and their title or it can be also expressed in text forms. The second table is the grading of CTTS stained cells of cancer and gastritis both or only tumor cells, not clear. In the demographic data table there are CTTS stained cells in both cancer and gastritis groups but in methods, you mentioned the grading of tumor cells.

**Answer: We have removed the tables of the Methods section and put the information included in text format.**

**The second table of IHC included both groups, gastritis and adenocarcinoma.**

**In the demographic.**

**In the demographic table, we included grading and expression of both groups. We have added this information in the Methods section.**

Results

1. Table 1 title. Comparison between gastric cancer and gastritis groups (demographic data)

**Answer: We have adjusted Table 1 title.**

2. Table 1, Percentage of CTTS stained cells. In the methods, the percentage has been evaluated for cancer cells only but here in gastritis also. It needs clarification

**Answer: We have adjusted this information in our Methods section.**

3. Paragraph 3. The explanation in the paragraph does not match Table 3. Table 3 does not contain characteristics of gastric adenocarcinoma.

**Answer: We have adjusted the paragraph according to Table 3. Thank you for pointing.**

4. Paragraph 4. The explanation in paragraph and figure 1 legend does not corroborate. In the paragraph, it is mentioned gastritis whereas figure 1 legend depicts gastric adenocarcinoma.

**Answer: We have adjusted the mentioned paragraph and figure. Thank you for pointing. We have changed figures order.**

5. Paragraph 5. Similar to the above comment, the explanation in paragraph 5 and figure 2 legend does not corroborate. In the paragraph, it is explained about gastric cancer and figure 2 legend it is about gastritis.

**Answer: We have adjusted the mentioned paragraph and figure. Thank you for pointing. We have changed figures order.**

Discussions

1. Cathepsins that have previously………….. are B, E, K, L, S, X, and Z. Provide references

**Answer: Reference #8 (Chen S, Dong H, Yang S, Guo H. Cathepsins in digestive cancers. Oncotarget. 2017;8(25):41690-41700. doi:10.18632/oncotarget.16677).**

2. Paragraph 2, Last sentence. The results of these studies suggest………… Which studies you are referring is not clear.

**Answer: We are referring to the results of our study and the previously reported reference earlier in the same paragraph, which are Liu et al. [14].**

3. Provide full form when you are referring first time and then short form; for example, provide full form of TNM

**Answer: The TNM staging system is a standardized nomenclature for the local and systemic staging for malignant tumours. The full form is the so called TNM.**

Minor comments

1. Three terms; gastric tumour, gastric cancer, and gastric adenocarcinoma have been used. I suggest using either one and making it uniform.

**Answer: We opted to use only gastric adenocarcinoma now.**

2. Throughout the manuscript, H. pylori should be italic

**Answer: We have adjusted it.**

**My co-authors and I found the recommendations of the reviewers to be extremely valuable and by addressing their concerns the manuscript has been significantly improved and we now hope that the manuscript is acceptable for publication. Thank you.**
